# Compressed intracellular motility via non-uniform temporal sampling in dynamic optical coherence tomography

**DOI:** 10.1117/1.JBO.29.7.076002

**Published:** 2024-07-04

**Authors:** Amy L. Oldenburg, Pan Ji, Xiao Yu, Lin Yang

**Affiliations:** a University of North Carolina at Chapel Hill, Department of Physics and Astronomy, Chapel Hill, North Carolina, United States; b University of North Carolina at Chapel Hill, Biomedical Research Imaging Center, Chapel Hill, North Carolina, United States

**Keywords:** optical coherence tomography, motility, dynamic optical coherence tomography, speckle fluctuation, autocorrelation

## Abstract

**Significance:**

Optical coherence tomography has great utility for capturing dynamic processes, but such applications are particularly data-intensive. Samples such as biological tissues exhibit temporal features at varying time scales, which makes data reduction challenging.

**Aim:**

We propose a method for capturing short- and long-term correlations of a sample in a compressed way using non-uniform temporal sampling to reduce scan time and memory overhead.

**Approach:**

The proposed method separates the relative contributions of white noise, fluctuating features, and stationary features. The method is demonstrated on mammary epithelial cell spheroids in three-dimensional culture for capturing intracellular motility without loss of signal integrity.

**Results:**

Results show that the spatial patterns of motility are preserved and that hypothesis tests of spheroids treated with blebbistatin, a motor protein inhibitor, are unchanged with up to eightfold compression.

**Conclusions:**

The ability to measure short- and long-term correlations compressively will enable new applications in (3+1)D imaging and high-throughput screening.

## 1 Introduction

By merit of its high speed and depth resolution, optical coherence tomography (OCT) has become a powerful tool for imaging dynamic processes, such as diffusion,^1^ flow,^2^ and cellular processes.^3^ A key benefit of dynamic OCT is the ability to distinguish biological processes by features of their temporal signals and associated statistics. For example, blood flow detection is improved by separation from bulk motion using a split spectrum analysis technique.^4^ One can distinguish cellular responses to different drugs by extracting features from speckle fluctuation spectra, as shown using a related low-coherence imaging technique.^5^ In OCT studies of ciliated tissues, one can detect ciliated surfaces and assess the ciliary beat frequency,^6^^,^^7^ or one might measure the diffusion rate of nanoparticles with OCT to image differences in tissue nanoporosity.^8^ Often, it is desired to contrast these dynamic features volumetrically, such as for quantitative retinal blood flow imaging,^9^ airway tissue surface dynamics,^10^ and imaging of organoid motility,^11^ which require high-dimensional (3+1)D imaging. Even if only cross-sectional (2+1)D imaging is needed, screening applications such as personalizing cancer chemotherapies^12^ and toxicant assays^13^ require additional dimensions of study conditions. The development of “compressed” sampling techniques would be beneficial for reducing OCT scanning requirements and memory overhead and favorable toward such high-throughput applications. While prior efforts have made progress in this regard using traditional “compressed sensing” (CS) methods in space,^14^ we introduce a “compressed” or sub-sampling method (that is not based on CS theory) along the temporal one dimension in such a way that temporal features of interest are preserved; we note that, unlike CS methods, we do not aim to reconstruct the fully sampled time signal but to reconstruct the temporal feature of intracellular motility, as defined in Ref. 15. To accomplish this, we propose a non-uniform temporal sampling (NUTS) method that estimates both short- and long-term correlations of the sample and yields accurate signal estimates at each point in space from highly under-sampled measurements. In comparison, uniform temporal sub-sampling (UTS) has been proposed^16^ with some success and compatibility with scanning protocols that can be implemented in hardware; however, simply reducing the sampling rate loses the ability to capture short-term correlations accurately. NUTS was notably proposed in the context of velocimetry,^17^ but the scheme employed a random selection of time points that are not readily implemented in hardware, and it was optimized solely for flow estimation. As shown below, the NUTS method proposed here strikes a balance between accurate estimation of both short- and long-term correlations while employing a regular sampling pattern that is readily implemented in hardware.

In this paper, we have framed the problem as that of estimating an OCT temporal autocorrelation-based intracellular motility metric, “motility amplitude” M, with temporal sub-sampling. The utility of M has been demonstrated in tracking the responses of human mammary epithelial cells (MECs) in three-dimensional (3D) cultures to drugs and toxicants.^13^^,^^15^^,^^18^^,^^19^ Below, we describe how M quantifies the relative amplitude of fluctuations exhibiting short-term correlations that are distinct from white noise and stationary features. Similar autocorrelation-based metrics for cellular motility contrast have been employed to assess corneal cross-linking^20^ and urothelial cancer cell distribution.^21^ Here, our NUTS method is applied to estimating M from the magnitude of the OCT signal, avoiding the need for phase-resolved OCT. However, the proposed method may also be applicable for compressively estimating decorrelations in the complex OCT signal (see, e.g., Ref. 22 for a discussion of differences between amplitude and phase-resolved methods). As shown below, the proposed NUTS method provides better estimation than uniform sampling under the same compression ratio, with the possibility of viable compression ratios of up to eight, which may dramatically improve scan time and storage efficiency in high throughput experiments.

## 2 Background and Methods

### 2.1 Distinguishing Dynamic Processes in OCT

**Fig. 1:**
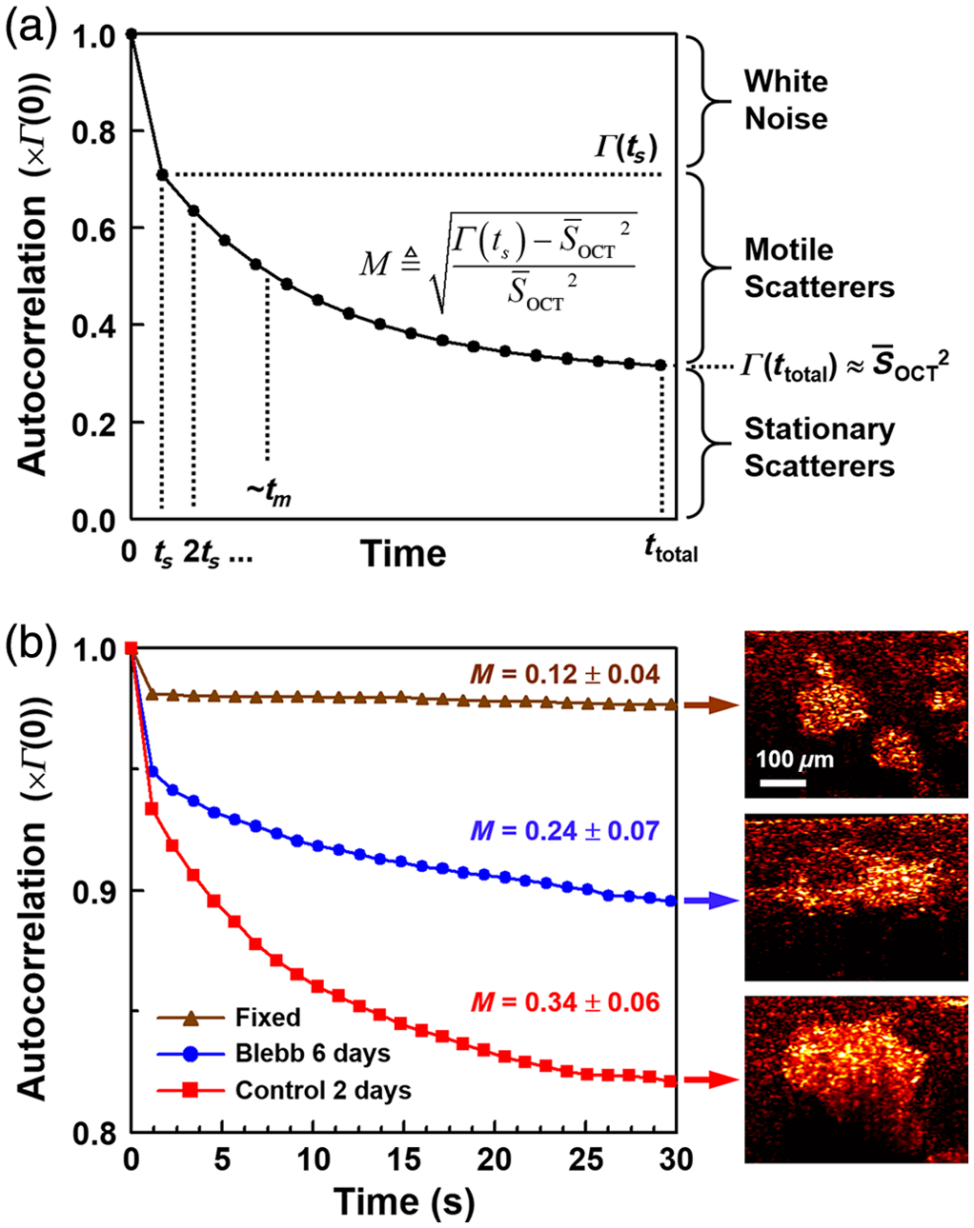
Short- and long-term processes visualized by temporal autocorrelations of the OCT signal, $S_{\mathrm{OCT}}$. Autocorrelations are displayed in units of Γ(0). (a) Idealized autocorrelation showing relative contributions of white noise, motile scatterers with characteristic decay time $t_{m}$, and stationary scatterers. The motility amplitude, M, isolates the contribution of motile scatterers. (b) Example autocorrelations from OCT of spheroids with low M (formalin-fixed), moderate M (blebbistatin treated), and high M (untreated). OCT videos for each condition can be seen in Video 1 (20× real-time) (Video 1, MP4, 3.40 MB [URL: https://doi.org/10.1117/1.JBO.29.7.076002.s1]).

We consider the time-varying magnitude of the OCT signal, $S_{\mathrm{OCT}}$, collected from a coherence volume in the single-scattering approximation. Within the coherence volume, there may be multiple light scatterers that contribute to $S_{\mathrm{OCT}}$, each moving with different velocities and different amounts of coordination. Let us consider collecting $S_{\mathrm{OCT}}$ with sampling time $t_{s}$ over a total time $t_{\text{total}}$, then computing its temporal autocorrelation Γ(t). An idealized autocorrelation trace is shown in Fig. 1(a). The autocorrelation trace primarily reveals processes that cause $S_{\mathrm{OCT}}$ to fluctuate on a time scale longer than $t_{s}$ but shorter than $t_{\text{total}}$; we call the time scale of such processes the memory time of the “motile” scatterers, $t_{m}$. Slowly moving scatterers that do not change $S_{\mathrm{OCT}}$ over $t_{\text{total}}$ gives rise to the autocorrelation at $t_{\text{total}}$, $\Gamma (t_{\text{total}})$. If we assume that these slow-moving scatterers are perfectly stationary, the autocovariance of $S_{\mathrm{OCT}}$ at $t_{\text{total}}$ is zero, and we may approximate $\Gamma (t_{\text{total}})$ as being equal to ${\overset{¯}{S}}_{\mathrm{OCT}}^{2}$. We consider an OCT system that is limited by white noise (e.g., shot noise, which is the limiting type of noise in many OCT systems). Because white noise has infinite bandwidth, it decorrelates instantaneously. Thus, the portion of the autocorrelation that decorrelates between the first two samples, $\Gamma (0)-\Gamma (t_{s})$, is imparted by white noise and by any other processes causing fluctuations faster than $t_{s}$ (e.g., extremely rapid-moving scatterers, as well as motile scatterers that exhibit a small amount of decorrelation over $t_{s}$). In this picture, we can isolate the contribution of the motile scatterers by approximately $\Gamma (t_{s})-{\overset{¯}{S}}_{\mathrm{OCT}}^{2}$, i.e., the amount that the autocorrelation trace decays between $t_{s}$ and $t_{\text{total}}$.

A few things should be noted at this stage. First, the difference $\Gamma (t_{s})-{\overset{¯}{S}}_{\mathrm{OCT}}^{2}$ is equivalent to the autocovariance. For discussion purposes, we chose to separate Γ (autocorrelation) and ${\overset{¯}{S}}_{\mathrm{OCT}}^{2}$, which are impacted differently by different sampling methods. Second, it may be possible to estimate the white noise contribution to Γ(0) using methods described in Ref. 23, which could provide a better estimate of the motile scatterer contribution. However, these methods were unable to be implemented in this study, as they require an OCT system with certain noise statistics and a good estimate of the signal-to-noise ratio (SNR).

To validate the picture of Fig. 1(a), example OCT data sets of MEC spheroids in 3D culture under varying conditions were analyzed. MEC spheroids exhibit intracellular motility (i.e., in-place motions of sub-resolution, intracellular components) that decorrelates OCT signals with a 1/e decay time on the order of 5 s.^24^ Here, we compared spheroids that were formalin-fixed and expected to exhibit no motility; spheroids that were treated with blebbistatin, a myosin II inhibitor, for 6 days and expected to exhibit suppressed motility; and spheroids that were untreated for 2 days as an example of high motility. (A shorter culture time point of 2 days for the latter was chosen to avoid possible apoptosis.) Pixelwise autocorrelation traces averaged over regions of the spheroids are displayed in Fig. 1(b). All traces exhibit an initial decay at $\Gamma (t_{s})$ between 2% and 7% of Γ(0) attributed predominantly to white noise. The trace of the fixed spheroid exhibits negligible further decay, consistent with the picture that it is comprised of stationary scatterers. By contrast, the traces of the blebbistatin and control spheroids decay further, reaching stable values at ∼30  s, suggesting that they contain motile scatterers. The blebbistatin-treated spheroid exhibits a smaller proportion of decay compared with the control, consistent with the expectation that it has suppressed motility (fewer and/or slower motile scatterers). In all cases, a significant fraction of the autocorrelation (between 83% and 99%) remains after $t_{\text{total}}=112\;s$ (not shown), which is attributed to stationary scatterers.

To estimate the contribution of the motile scatterers in a manner that is independent of both white noise and the signal level ${\overset{¯}{S}}_{\mathrm{OCT}}$, we previously proposed a motility amplitude metric M according to Ref. 15

$$M=\sqrt{\frac{\Gamma (t_{s})-{\overset{¯}{S}}_{\mathrm{OCT}}^{2}}{{\overset{¯}{S}}_{\mathrm{OCT}}^{2}}}.$$ (1)

Looking at Fig. 1(a), we can see how M quantifies the motile scatterer contribution and is normalized by ${\overset{¯}{S}}_{\mathrm{OCT}}^{2}$ such that it is a unitless number; the final square root linearizes M with respect to the OCT signal. This choice of normalization was found to impart depth-invariance to M, even under significant OCT signal roll-off.^15^ The value of M is reported for each of the spheroid conditions displayed in Fig. 1(b), which ranges from a background level of 0.12 for the fixed spheroid, to a high value of 0.34 for the control spheroid. Thus, while long-term correlations are quantified by the time-averaged OCT signal ${\overset{¯}{S}}_{\mathrm{OCT}}$, M is an excellent proxy for the relative contribution of short-term ($t_{s}<t_{m}<t_{\text{total}}$) correlations in dynamic OCT data. As such, here, we focus our efforts on compressive sampling toward accurately reconstructing “motility images” rendered by M.

### 2.2 Proposed Method of Compressive Sampling

Our goal is to collect fewer samples of $S_{\mathrm{OCT}}$ while maintaining the ability to accurately estimate the short-term correlation, $\Gamma (t_{s})$, and the long-term correlation, $\Gamma (t_{\text{total}})\approx {\overset{¯}{S}}_{\mathrm{OCT}}^{2}$, which subsequently enables computation of M according to Eq. (1). Given the unbiased, discrete autocorrelation of $S_{\mathrm{OCT}}$

$$\Gamma (j\cdot t_{s})=\frac{1}{N-j}\overset{N-j-1}{\underset{i=0}{\sum }}S_{\mathrm{OCT}}((i+j)t_{s})S_{\mathrm{OCT}}(i\cdot t_{s}),$$ (2)

where N is the number of samples; we write $\Gamma (t_{s})$ as

$$\Gamma (t_{s})=\frac{1}{N-1}\overset{N-2}{\underset{i=0}{\sum }}S_{\mathrm{OCT}}((i+1)t_{s})S_{\mathrm{OCT}}(i\cdot t_{s}).$$ (3)

Given a maximum available sampling rate of the OCT system $1/t_{s}$, we see that it is necessary to collect adjacent pairs of $S_{\mathrm{OCT}}$ samples ($i\cdot t_{s},(i+1)t_{s}$), to compute $\Gamma (t_{s})$. We might consider simply collecting fewer samples N; however, that would decrease $t_{\text{total}}$, lead to a smaller time window for $t_{m}$ of the motile processes, and rapidly break down the approximation $\Gamma (t_{\text{total}})\approx {\overset{¯}{S}}_{\mathrm{OCT}}^{2}$, which, based on simulations presented below, is likely to already be a limiting factor in our experiments. If, on the other hand, we increase the sampling time $t_{s}$ while keeping $t_{\text{total}}$ constant, we similarly narrow the available window for $t_{m}$ on the short time side and potentially lose motility signal amplitude for portions of the signal that decay faster than the longer $t_{s}$. In the latter scenario, we will call the UTS method, where $t_{s}$ is increased by some integer compression ratio, e.g., $t_{s}\to 2t_{s}$, $4t_{s}$, or $8t_{s}$ (and N→N/2, N/4, or N/8) for twofold, fourfold, or eightfold compression, respectively. In the UTS method, the expression of Eq. (3) is used to compute $\Gamma (t_{s})$, and the mean of all samples is computed to estimate ${\overset{¯}{S}}_{\mathrm{OCT}}$.

Instead of UTS, here, we propose a non-uniform (NUTS) method that avoids narrowing the window of $t_{m}$. This is accomplished by collecting adjacent pairs of samples (separated only by $t_{s}$) with an appropriate intervening dead time. Given a desired compression ratio r, we write the sampling scheme as

(i,i+1)=(0,1),(2r,2r+1),(4r,4r+1),  and  (6r,6r+1). (4)

For example, to achieve a compression ratio of r=4, the signal would be sampled at times $\left(0,t_{s},8t_{s},9t_{s},16t_{s},17t_{s}\ldots \right)$. In the NUTS method, $\Gamma (t_{s})$ is then computed by multiplying the adjacent pairs of samples according to

$$\Gamma (t_{s})=\frac{1}{N^{\prime }}\overset{N-2}{\underset{i=0,2r,4r,\ldots }{\sum }}S_{\mathrm{OCT}}((i+1)t_{s})S_{\mathrm{OCT}}(i\cdot t_{s}),$$ (5)

where N is the number of samples if the system was uncompressed [i.e., $N=(t_{\text{total}}/t_{s})+1$] and $N^{\prime }$ is the number of compressively sampled pairs (which ≈N/2r with rounding). As with the UTS method, ${\overset{¯}{S}}_{\mathrm{OCT}}$ in NUTS is estimated by computing the mean of all available samples. We note that, while not used in this paper, if one wished to estimate the variance of $\Gamma (t_{s})$, one would employ the finite population correction since $\Gamma (t_{s})$ is sampled without replacement.

### 2.3 Monte Carlo Simulations

To quantify the impact of NUTS on extracting motility data from OCT images, we developed a simple simulation model to capture the additive contributions of white noise, motile scatterers, and stationary scatterers. The OCT signal sampled at N evenly spaced time points, $t_{i}$, i=0…,N−1, is written as follows:

$$S_{\mathrm{OCT}}(t_{i})=c_{n}f_{n}(t_{i})+c_{m}f_{m}(t_{i})+1,$$ (6)

where $f_{n}$ is the contribution of white noise, $f_{m}$ is the contribution of motile scatterers with a persistence (memory) time of p sampling points, and an offset of 1 represents the stationary scatterers. The weights $c_{m}$ and $c_{n}$ are used to adjust the contributions of motile scatterers and white noise, respectively, relative to the offset. (For simplicity, here we assume that noise is purely additive, and we ignore contributions from multiplicative noise; the latter was found to have negligible impact at the noise levels explored in this paper.) The white noise and motility functions are computed according to

$$f_{n}(t_{i})=N(0,\;1),$$ (7)

and

$$f_{m}(t_{i})=\sqrt{p}(\frac{1}{p}\overset{i+p-1}{\underset{j=i}{\sum }}X(t_{j}));\;X(t_{j})=N(0,\;1),$$ (8)

respectively, where N(0,1) denotes a zero-mean normal distribution with a variance of 1. While the white noise is represented by a purely random distribution, the motile scatterers are represented by a function with memory that is imparted by taking a moving average over a time window of $p\cdot t_{s}$. An additional weight of $\sqrt{p}$ is introduced to keep the variance of $f_{m}$ constant at 1. While there are other models for memory that could be used, such as diffusion, for the purposes of this study, we do not wish to presume a particular physical model for the dynamic process. We should note a few things about our choice of a normal distribution for these simulations. First, owing to the third term offset of Eq. (6), the net $S_{\mathrm{OCT}}$ signal has statistics of a normal distribution with a non-zero mean. The adjustability of $c_{n}$ relative to this offset effectively simulates pixels of the same SNR. There are other distributions more commonly used to describe *spatial* speckle statistics, such as the Rayleigh distribution. We found that upon analyzing the *temporal* statistics of our experimental data, there was no significant difference in $R^{2}$ values when fitting to Rayleigh or normal distributions for pixels of similar mean values (and thus similar SNRs), with typical $R^{2}$ values of 0.99. Given the relative simplicity and ubiquity of the normal distribution, we chose to perform simulations under this assumption.

Importantly, the model of Eqs. (6)–(8) allows us to test the impact of varying contributions of white noise, motile scatterers, and stationary scatterers. Because the variances of the noise and motile scatterer contributions are scaled by $c_{n}^{2}$ and $c_{m}^{2}$, respectively, and ${\overset{¯}{S}}_{\mathrm{OCT}}$ is given by the offset, which is equal to 1, we expect that

$$\Gamma (0)=c_{n}^{2}+c_{m}^{2}+1,$$ (9)

$$\Gamma (t_{s})\approx c_{m}^{2}+1,$$ (10)

$$\Gamma (t_{\text{total}})\approx {\overset{¯}{S}}_{\mathrm{OCT}}^{2}=1,$$ (11)

and thus, M according to Eq. (1) is approximately equal to $c_{m}$.

### 2.4 Mammary Spheroid 3D Cultures

3D cell cultures were prepared as described previously in Ref. 19. The data analyzed and compressively sampled here are a subset of those initially reported in that paper. Briefly, the spheroids studied here comprised MCF10DCIS.com cells, a pre-malignant human MEC line that forms ductal carcinoma in situ (DCIS)-like lesions in xenograft models.^25^ Cells were initially cultured in 2D until reaching 70% confluency and then seeded in 3D at a density of $30,000\text{ cells}/{\mathrm{cm}}^{3}$ into an artificial extracellular matrix comprised of 1:1 collagen I:Matrigel (BD Biosciences, Franklin Lakes, New Jersey, United States) at a final collagen I concentration of 1  mg/mL. The MCF10DCIS.com cells were subsequently grown for 10 days to allow for spheroid formation in the 3D cultures. At this time, blebbistatin, an inhibitor of the motor protein myosin II, was introduced into the cell media at either 0, 25, or 50  μM concentration. OCT imaging was performed on cultures immediately prior to blebbistatin exposure and subsequently at 1 h, 24 h, 48 h, and 6 days post-exposure, for a total of 15 unique dose and time conditions.

For images of fixed spheroids reported in Fig. 1, 3D cultures were prepared as above, grown for 19 days, and then fixed with 10% v/v formalin.

### 2.5 OCT Imaging and Post-Processing

3D organoid cultures were imaged by a custom spectral-domain OCT system described in detail previously,^26^ using the methods specifically outlined in Ref. 19. Briefly, the light source is a Ti:Sapphire laser with a central wavelength of 800 nm and bandwidth of 120 nm, providing a lateral and axial resolution of ∼10  μm×3  μm (in aqueous medium), respectively, and ∼6  mW of optical power at the sample. The system exhibits a sensitivity of ∼108  dB under the conditions used in this study. Polarization control was used such that the sample was illuminated with horizontally polarized light, and returned backscattered light was passed through a polarizer to select the co-polarized (horizontal) component; this is important since temporal dynamics in OCT may differ between co- and cross-polarized components.^8^ Images were collected into 1000×1024  pixels over 3  mm×1.5  mm (in aqueous medium) laterally and axially, respectively. The A-line rate was set to 2 kHz, and the resulting frame rate (accounting for dead time between frames) was 0.89 Hz. A total of 300 B-mode images were collected (at even time intervals) and divided into 3 sets of N=100 images each for independent analysis (where uneven time intervals were achieved by sub-sampling these data). As such, we expect this sampling condition to allow for the detection of motile scatterers with fluctuations on time scales between $t_{s}=1.12\;s$ and $t_{\text{total}}=112\;s$.

#### 2.5.1 Experimental image post-processing

Raw spectral OCT data were converted to depth-resolved B-mode images via Fourier transformation using digital dispersion compensation^27^ and reference subtraction. The region of each visible spheroid was segmented with assistance by a custom script to semi-automate the process,^28^ with typically one to four spheroids per B-mode image set. M was computed at each pixel according to methods outlined in Sec. 2.2. For uncompressed analyses, all N=100 images were used, while for compressive sampling, a subset of images was selected as described above. Both NUTS and UTS methods were employed using compression ratios of 2, 4, 8, and 16, reducing the total number of samples for UTS to N=50, 25, 13, and 7, respectively. With NUTS, the total number of samples was reduced to $2N^{\prime }=50$ (25 pairs), 26 (13 pairs), 14 (7 pairs), and 8 (4 pairs), via the method of Eq. (4). While the total number of retained samples is slightly better for many of the non-uniformly sampled sets, as will be shown below, the overall comparative performances show much larger trends than can be explained by these differences.

The accuracy of M estimation was evaluated on a pixel-by-pixel and spheroid-by-spheroid basis. The pixel-wise M reveals the spatial pattern of motility inside spheroids. In the pixel-wise M analysis, a spatial mean filter of 12  μm×3  μm (4×2  pixels) window size (laterally and axially, respectively) was applied to uncompressed and compressed M images. Subsequent segmentation of the spheroid regions was used to produce pixel-wise scatter plots of compressed versus uncompressed M. Scatter plots were fitted to a regression line of the form y=mx (no offset), and the slope m and correlation coefficient (Pearson’s r) were computed. Spheroid-averaged M, computed by segmenting M images without any prior mean filtering, was used to track the motility response of spheroids to different culture conditions (toxicant dose and exposure time). M was computed uncompressed and via NUTS and UTS with fourfold and eightfold compression. Spheroid-averaged scatter plots comparing compressed to uncompressed M were analyzed as before. Then, M data grouped by culture condition were analyzed in parallel for each method (uncompressed and fourfold and eightfold NUTS and UTS), performing a t-test (two-tailed, heteroscedastic) comparing M data of spheroids at each dose and time condition to the corresponding M data before exposure.

#### 2.5.2 Simulation data processing

The Monte Carlo model described in Sec. 2.3 was employed to simulate the dynamic OCT signal collected within a pixel using settings chosen to match experimental conditions. As such, each simulation for a particular input of noise and motile scatterer weights $c_{n}$ and $c_{m}$ was comprised of a time series of N=100 points and repeated 6000 times (to approximate the average number of pixels within the spheroids in our study). M for each of the 6000 simulated time series was computed and averaged in the same way as for experimental OCT data to produce the simulated spheroid-averaged M.

## 3 Results and Discussion

### 3.1 Simulations of Compressive Sampling

**Fig. 2:**
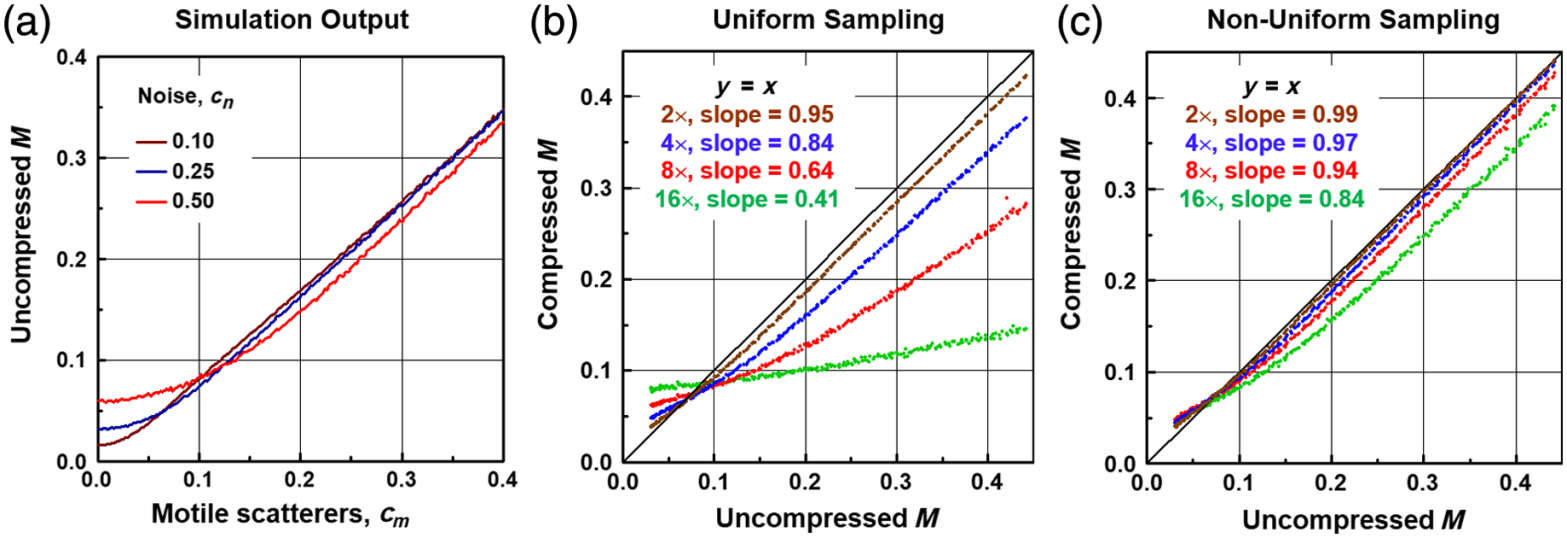
Monte Carlo simulations of dynamic OCT signals using the model of Eq. (6). (a) Simulated measurements of M with varying contributions of motile scatterers ($c_{m}$) and noise ($c_{n}$). As noise increases, the background M level increases. (b), (c) Scatter plots of simulated M measurements (compressed versus uncompressed) with uniform or NUTS, respectively. While all conditions exhibited a good correlation between compressed and uncompressed M, as the compression ratio increased, the slopes obtained by linear regression decreased from the ideal value of 1. Overall, the proposed method of non-uniform sampling exhibited superior performance, with a slope of 0.94 at eightfold compression. In these simulations, $c_{m}$ was varied from 0 to 0.5, and $c_{n}$ was set to 0.25 to approximate typical spheroid data.

The model of Eq. (6) was employed to systematically investigate the impact of the proposed NUTS method on measurements of M spatially averaged over a typical spheroid size. Figure 2(a) shows how M is varied in this model by modifying the weight of the motility function, $c_{m}$, under varying levels of noise, $c_{n}$. As expected, M nearly equals $c_{m}$ and increases monotonically with it. There is a threshold minimum value of M as $c_{m}$ approaches zero that increases with increasing noise, which is important to note when interpreting M from samples with predominantly stationary scatterers or regions with low SNR. The fact that M is somewhat smaller than $c_{m}$ even for large input values is due to the approximative nature of Eqs. (10) and (11), i.e., that some of the motile scatterer contributions decorrelate before $t_{s}$, and some of them do not decorrelate by $t_{\text{total}}$, giving rise to an under-estimate of $\Gamma (t_{s})$ and over-estimate of ${\overset{¯}{S}}_{\mathrm{OCT}}^{2}$, both of which reduce M via Eq. (1). The amount of these errors depends on the exact choice of the memory time, and details are given in Fig. S1 in the Supplementary Material. For the simulations of Fig. 2, we chose a memory time of $t_{m}=20$ $t_{s}$ (p=20) and the total number of samples N=100 to recapitulate the relative values of $t_{m}$, $t_{s}$, and $t_{\text{total}}$ in MEC spheroid experiments. Notably, the simulations of Fig. S1 in the Supplementary Material show that, for p=20, the error in M arising from ${\overset{¯}{S}}_{\mathrm{OCT}}^{2}$ is significantly larger than that from $\Gamma (t_{s})$, although we were unable to confirm this trend in the experimental data reported below as we lacked ground-truth values of M.

It is interesting to note that the reduction of M relative to $c_{m}$ could be corrected if one knew *a priori* the functional form of the autocorrelation decay curve, such as an inverse exponential in the case of diffusing scatterers. However, a diffusion model, which would be consistent with a Lorentzian power spectrum, is inconsistent with power spectra from MEC spheroids in other experiments.^15^ Therefore, such an approach may not be possible for the highly complex MEC spheroids of this study.

Figures 2(b) and 2(c) illustrate the impact of uniform compressive sampling (i.e., by simply increasing $t_{s}$) and the proposed NUTS method of Eq. (4), respectively. Each point on the scatter plots represents a given simulation where M was computed either from the full data (uncompressed) or by appropriately sub-sampling the data (compressed). We can clearly see a rapid degradation in the accuracy of M estimation with increasing compression ratio for the uniform sampling method, where M above ∼0.1 is significantly under-estimated for compression ratios of 4 and above (slope≤0.84). This is expected because $t_{s}$ increases with increasing compression, resulting in increased decorrelation by the motile scatterers [breakdown of Eq. (10), which lowers the overall M value]. By contrast, for the proposed non-uniform sampling method, M is well reproduced at up to eightfold compression, where it keeps ∼94% of its baseline value. With NUTS, we found that the breakdown of Eq. (11) (inaccurate estimation of ${\overset{¯}{S}}_{\mathrm{OCT}}^{2}$) ultimately led to the reduced slope relative to the ideal y=x line.

### 3.2 NUTS Preserves Spatial Patterns of Intracellular Motility

**Fig. 3:**
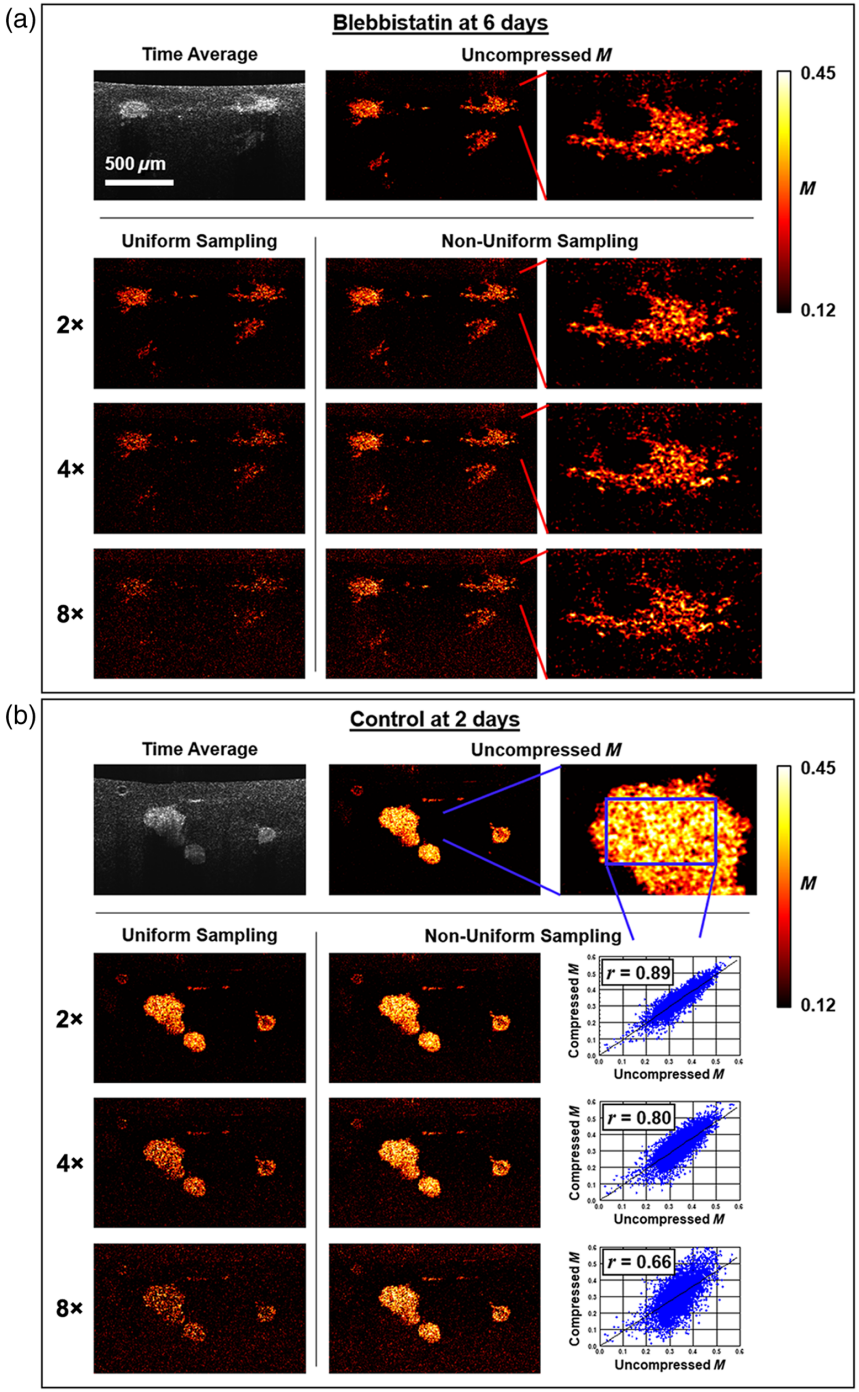
(a), (b) Effects of compressive sampling on motility imaging of weakly motile spheroids (blebbistatin-treated) and highly motile spheroids (control), respectively. Top row: Time-averaged OCT images and corresponding uncompressed motility images. Subsequent rows show the effects of increased compression ratio (2×, 4×, and 8×) with either uniformly sampled data (left) or the proposed NUTS method (middle) for each condition. (a, right column) Closeup of a blebbistatin-treated spheroid with varying levels of NUTS, showing how the structure is preserved. (b, right column) Pixel-by-pixel scatter plots of non-uniformly compressively sampled versus uncompressed M for a control spheroid demonstrate a good-to-moderate correlation r with increasing compression.

Given the promising results of the simulations, we next explore how the proposed NUTS method performs on an existing dataset of MEC spheroids collected without compression, with UTS also performed as a point of comparison. Motility images, i.e., spatial maps of M computed at each pixel and subsequently mean filtered over a resolution window, are computed for the two examples of live spheroids in Fig. 1. Motility images computed under varying levels of compression are displayed in Fig. 3, with the time-averaged OCT images (${\overset{¯}{S}}_{\mathrm{OCT}}$) displayed in grayscale for reference. As expected, we can qualitatively see that while ${\overset{¯}{S}}_{\mathrm{OCT}}$ exhibits heavy attenuation with depth, the motility images do not suffer from signal roll-off. Importantly, consistent with the simulations, we see that the motility images using UTS decrease in contrast dramatically to the increasing compression ratio, while NUTS maintains both the contrast and spatial pattern of M up to eightfold compression. Particularly telling is a closeup of one spheroid in the blebbistatin-treated sample with a complex structure that is maintained after NUTS. These findings are favorable for future investigations into spatial patterns of motility within spheroids as related to drug or toxicant exposures and associated cell responses such as cancer metastasis.

To quantify the accuracy of NUTS, the right column of Fig. 3(b) shows pixel-by-pixel scatter plots of compressed versus uncompressed M within a region of interest inside a control spheroid. Consistent with simulations, the slope of the regression line decreases with increasing compression from 0.985 (twofold) to 0.913 (eightfold). The spread of the data, however, appears qualitatively larger than in simulations, an effect which can be quantified by Pearson’s r. We found that Pearson’s r decreases with increasing compression from 0.89 (twofold) to 0.66 (eightfold). To show how the NUTS method performs across the entire data set, which represents a variety of dose and culture time conditions, we tabulated the slope and Pearson’s r computed from pixels within each spheroid and then averaged across each unique culture condition (see Table S1 in the Supplementary Material). Overall, the findings are similar to those represented in Fig. 3, with little change in accuracy as a function of culture condition.

### 3.3 NUTS Preserves Results of Hypothesis Testing

**Fig. 4:**
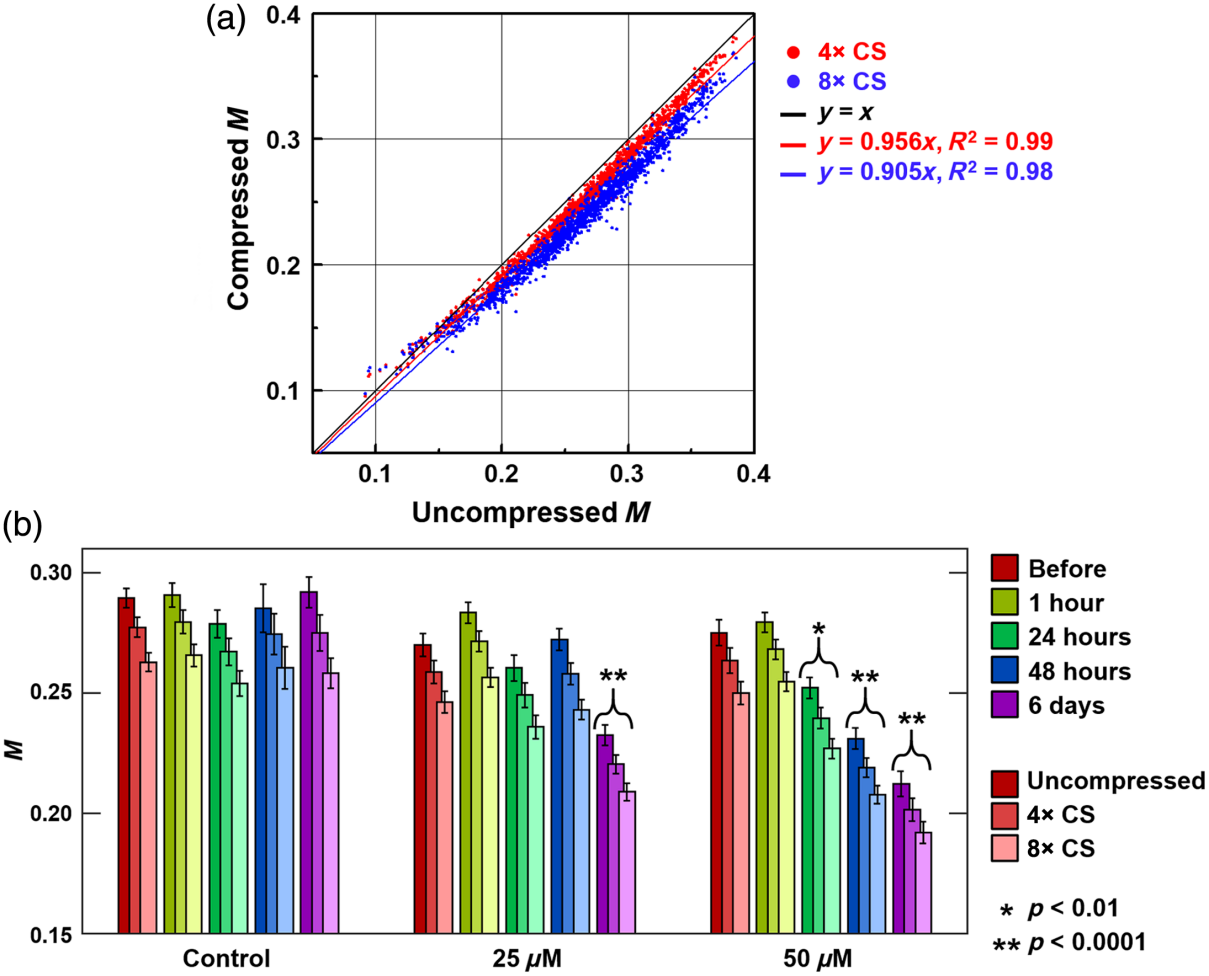
(a) Spheroid-by-spheroid scatter plot of 4× and 8× non-uniformly compressively sampled versus uncompressed M overall blebbistatin concentrations and time points (n=1428). Best-fit lines indicate good linearity with a small reduction in the slope from the ideal y=x line as the compression level is increased from 4× to 8×. (b) Dose- and time-dependent M (mean ± std. err) of spheroids exposed to blebbistatin under varying compression levels. Hypothesis testing (two-tailed t-test compared with corresponding before values) indicates similar significance levels when performed on compressed or uncompressed data.

Because temporal dynamics of *in vitro* cell cultures imaged by OCT are increasingly used to infer responses of cells to a variety of perturbations, it is important to ensure that compressively sampled OCT does not alter the conclusions drawn from such experiments. Here, we tested the veracity of the proposed NUTS method to assess the significance of changes in MEC spheroid intracellular motility in response to blebbistatin exposure. A data set of MEC spheroids imaged in triplicate with OCT for a total of n=1428 was used for this purpose. Previously, spheroid-averaged M was computed and shown to be significantly different before and after exposure for certain blebbistatin doses and time points.^19^ Here, we non-uniformly sub-sampled these data according to Eq. (4), computed M according to Eq. (5), and performed the same hypothesis tests with the compressed M values to look for any differences in statistical significance. The results are summarized in Fig. 4. As a point of comparison, the results when applying UTS are displayed in Fig. S2 in the Supplementary Material.

A spheroid-to-spheroid scatter plot of non-uniformly compressively sampled versus uncompressed M across this data set is displayed in Fig. 4(a). Similar to pixel-to-pixel data, the regression line slope decreases with increasing compression. However, the spread of data is significantly smaller for spheroid-averaged data due to increased spatial averaging, exhibiting high correlations ($R^{2}=0.99$ and 0.98 for fourfold and eightfold compression, respectively). The results also display the same trends as those of the simulations displayed in Fig. 2(c). Figure 4(b) shows the resulting condition-averaged M for each dose and time point under each compression level. As shown, the overall M values become smaller with higher compression (as evidenced by the decrease in the slope of the scatter plot regression line from the ideal value of 1 to 0.956 and 0.905 for fourfold and eightfold compression, respectively). However, because the M values within each compression level track together (i.e., have a high correlation), the associated p-values remain largely unchanged (see Table S2 in the Supplementary Material), and the same results when examining p-values relative to significance levels are obtained [see asterisks on the bar chart in Fig. 4(b)].

## 4 Conclusion

A method is proposed for compressive measurements of intracellular motility with OCT by non-uniformly sampling the OCT data in time to capture short- and long-term correlations within the sample. Compared with uniformly sampling data in time, the NUTS method exhibits dramatically improved accuracy, both in simulation and on experimental data, for estimating the relative amplitude of fluctuations imparted by motile scatterers. Here, motile scatterers are defined as those that cause OCT signal fluctuations at time scales longer than the sampling time $t_{s}$ and shorter than the total imaging time $t_{\text{total}}$. When applying this technique to different applications, a careful choice of both $t_{s}$ and $t_{\text{total}}$ is first needed to define the temporal window over which motile scatterers are contrasted by this technique. It is also important to note that $t_{\text{total}}$ should be less than the time over which bulk motion causes significant speckle decorrelation, which may limit the applicability of this method to mechanically stabilized biological systems.

Our study shows that the data reduction levels achievable by NUTS are dramatic, with up to eightfold compression on a data set with 100 time samples preserving both the spatial pattern of intracellular motility in MEC spheroids and the statistical significance of cellular responses to blebbistatin treatment. This constitutes a leap forward in our capability to employ OCT as a non-invasive tool to study dynamic processes, which is currently limited by both scanning times and data overhead. For example, one could implement the sampling strategy of Eq. (6) by breaking a volume up into sub-volumes comprised of a set of r adjacent B-mode frames (where r is the compression ratio), cycling through each elevational position while pausing long enough at each to collect sequential frames, and returning back to each position in the same sequence over the desired total number of samples. Current commercially available OCT systems can be programmed to scan with such patterns, as well as OCT systems generally capable of BM- or MB-mode imaging. The concomitant reduction in data overhead is also crucial to reduce a typical OCT image stack for one cross-section in time from ∼100 to ∼13  MB in size, which is significant when multiplied over the number of frames in a volume for (3+1)D imaging. These enhancements will enable new applications in OCT-based sensing of 3D cell cultures in particular, where high-throughput imaging is desired for drug discovery and toxicant exposure applications. Laser speckle contrast imaging, which similarly involves the assessment of autocovariance of speckle fluctuations from biological media,^29^ may also benefit from the methods proposed here. The NUTS method may be broadly applicable to studying dynamic processes in complex biological or non-biological systems exhibiting temporal correlations on different time scales.

## Supplementary Material





## Data Availability

Some of the tabular data of this paper are available within the article or as Supplementary Material. The remaining data presented in this article, as well as an archived version of the code used in this paper, are publicly available in FigShare at https://doi.org/10.6084/m9.figshare.25605486. Additional, raw imaging data that were processed as part of this effort are available from the authors upon request.

## References

[1] Boas D. A. Bizheva K. K. Siegel A. M., “Using dynamic low-coherence interferometry to image Brownian motion within highly scattering media,” Opt. Lett. 23(5), 319–321 (1998). OPLEDP 0146-9592 10.1364/OL.23.000319 18084498

[2] Izatt J. A. et al., “In vivo bidirectional color Doppler flow imaging of picoliter blood volumes using optical coherence tomography,” Opt. Lett. 22(18), 1439–1441 (1997). OPLEDP 0146-9592 10.1364/OL.22.001439 18188263

[3] Farhat G. et al., “Detecting apoptosis using dynamic light scattering with optical coherence tomography,” J. Biomed. Opt. 16(7), 070505 (2011). JBOPFO 1083-3668 10.1117/1.3600770 21806246

[4] Jia Y. et al., “Split-spectrum amplitude-decorrelation angiography with optical coherence tomography,” Opt. Express 20(4), 4710–4725 (2012). OPEXFF 1094-4087 10.1364/OE.20.004710 22418228 PMC3381646

[5] Nolte D. D. et al., “Tissue dynamics spectroscopy for phenotypic profiling of drug effects in three-dimensional culture,” Biomed. Opt. Express 3(11), 2825–2841 (2012). BOEICL 2156-7085 10.1364/BOE.3.002825 23162721 PMC3493238

[6] Oldenburg A. L. et al., “Monitoring airway mucus flow and ciliary activity with optical coherence tomography,” Biomed. Opt. Express 3(9), 1978–1992 (2012). BOEICL 2156-7085 10.1364/BOE.3.001978 23024894 PMC3447542

[7] Chu K. K. et al., “In vivo imaging of airway cilia and mucus clearance with micro-optical coherence tomography,” Biomed. Opt. Express 7(7), 2494–2505 (2016). BOEICL 2156-7085 10.1364/BOE.7.002494 27446685 PMC4948609

[8] Chhetri R. K. et al., “Probing biological nanotopology via diffusion of weakly constrained plasmonic nanorods with optical coherence tomography,” Proc. Natl. Acad. Sci. U. S. A. 111(41), E4289–E4297 (2014). 10.1073/pnas.1409321111 25267619 PMC4205670

[9] Baumann B. et al., “Total retinal blood flow measurement with ultrahigh speed swept source/Fourier domain OCT,” Biomed. Opt. Express 2(6), 1539–1552 (2011). BOEICL 2156-7085 10.1364/BOE.2.001539 21698017 PMC3114222

[10] Münter M. et al., “Microscopic optical coherence tomography (mOCT) at 600 kHz for 4D volumetric imaging and dynamic contrast,” Biomed. Opt. Express 12(10), 6024–6039 (2021). BOEICL 2156-7085 10.1364/BOE.425001 34745719 PMC8547980

[11] Scholler J. et al., “Dynamic full-field optical coherence tomography: 3D live-imaging of retinal organoids,” Light: Sci. Appl. 9(1), 1–9 (2020). 10.1038/s41377-020-00375-8 32864115 PMC7429964

[12] Li Z. et al., “Intracellular optical Doppler phenotypes of chemosensitivity in human epithelial ovarian cancer,” Sci. Rep. 10(1), 17354 (2020). SRCEC3 2045-2322 10.1038/s41598-020-74336-x 33060663 PMC7562924

[13] Yu X. et al., “Quantification of the effect of toxicants on the intracellular kinetic energy and cross-sectional area of mammary epithelial organoids by OCT fluctuation spectroscopy,” Toxicol. Sci. 162(1), 234–240 (2017). 10.1093/toxsci/kfx245 PMC6059173 29140506

[14] Liu X. Kang J. U., “Compressive SD-OCT: the application of compressed sensing in spectral domain optical coherence tomography,” Opt. Express 18(21), 22010–22019 (2010). OPEXFF 1094-4087 10.1364/OE.18.022010 20941102 PMC3001297

[15] Oldenburg A. L. et al., “Inverse-power-law behavior of cellular motility reveals stromal–epithelial cell interactions in 3D co-culture by OCT fluctuation spectroscopy,” Optica 2(10), 877–885 (2015). 10.1364/OPTICA.2.000877 26973862 PMC4783137

[16] El-Sadek I. A. et al., “Three-dimensional dynamics optical coherence tomography for tumor spheroid evaluation,” Biomed. Opt. Express 12(11), 6844–6863 (2021). BOEICL 2156-7085 10.1364/BOE.440444 34858684 PMC8606131

[17] Mohan N. Vakoc B., “Principal-component-analysis-based estimation of blood flow velocities using optical coherence tomography intensity signals,” Opt. Lett. 36(11), 2068–2070 (2011). OPLEDP 0146-9592 10.1364/OL.36.002068 21633451 PMC3560399

[18] McIntosh J. C. et al., “Tracking the invasion of breast cancer cells in paper-based 3D cultures by OCT motility analysis,” Biomed. Opt. Express 11(6), 3181–3194 (2020). BOEICL 2156-7085 10.1364/BOE.382911 32637249 PMC7316000

[19] Yang L. et al., “Characterizing optical coherence tomography speckle fluctuation spectra of mammary organoids during suppression of intracellular motility,” Quant. Imaging Med. Surg. 10(1), 76 (2020). 10.21037/qims.2019.08.15 31956531 PMC6960418

[20] Blackburn B. J. et al., “Noninvasive assessment of corneal crosslinking with phase-decorrelation optical coherence tomography,” Invest. Ophthalmol. Vis. Sci. 60(1), 41–51 (2019). IOVSDA 0146-0404 10.1167/iovs.18-25535 30601930 PMC6322634

[21] Xu Z. Zhu H. Wang H., “Segmentation of the urothelium in optical coherence tomography images with dynamic contrast,” J. Biomed. Opt. 26(8), 086002 (2021). JBOPFO 1083-3668 10.1117/1.JBO.26.8.086002 34390233 PMC8363479

[22] Kalkman J. Sprik R. van Leeuwen T. G., “Path-length-resolved diffusive particle dynamics in spectral-domain optical coherence tomography,” Phys. Rev. Lett. 105(19), 198302 (2010). PRLTAO 0031-9007 10.1103/PhysRevLett.105.198302 21231201

[23] Uribe-Patarroyo N. et al., “Noise and bias in optical coherence tomography intensity signal decorrelation,” OSA Contin. 3(4), 709–741 (2020). 10.1364/OSAC.385431 34085035 PMC8171193

[24] Oldenburg A. L. et al., “Motility-, autocorrelation-, and polarization-sensitive optical coherence tomography discriminates cells and gold nanorods within 3D tissue cultures,” Opt. Lett. 38(15), 2923–2926 (2013). OPLEDP 0146-9592 10.1364/OL.38.002923 23903180 PMC3856705

[25] Miller F. R. et al., “MCF10DCIS.com xenograft model of human comedo ductal carcinoma in situ,” J. Natl. Cancer Inst. 92(14), 1185–1186 (2000). JNCIEQ 10.1093/jnci/92.14.1185a 10904098

[26] Chhetri R. K. et al., “Imaging three-dimensional rotational diffusion of plasmon resonant gold nanorods using polarization-sensitive optical coherence tomography,” Phys. Rev. E Stat. Nonlinear Soft Matter Phys. 83(4–1), 040903 (2011). 10.1103/PhysRevE.83.040903 PMC3116207 21599108

[27] Marks D. L. et al., “Digital algorithm for dispersion correction in optical coherence tomography for homogeneous and stratified media,” Appl. Opt. 42(2), 204–217 (2003). APOPAI 0003-6935 10.1364/AO.42.000204 12546500

[28] Fuller A. M. et al., “Epithelial p53 status modifies stromal-epithelial interactions during basal-like breast carcinogenesis,” J. Mammary Gland Biol. 26(2), 89–99 (2021). 10.1007/s10911-020-09477-w PMC8715550 33439408

[29] David A. B. Andrew K. D., “Laser speckle contrast imaging in biomedical optics,” J. Biomed. Opt. 15(1), 011109 (2010). JBOPFO 1083-3668 10.1117/1.3285504 20210435 PMC2816990

[30] Oldenburg A. L. et al., “Temporal non-uniform compressive sampling for dynamic optical coherence tomography” (accessed 2 January 2023).

