# Supplementary material for: Compressed intracellular motility via non-uniform temporal sampling in dynamic optical coherence tomography
Source: J Biomed Opt. 2024 Jul 4;29(7):076002. doi: 10.1117/1.JBO.29.7.076002 (PMC11223688; doi:10.1117/1.JBO.29.7.076002)
Supplement: Supplementary file 1 [file JBO_029_076002_SD001.pdf]

Compressed Intracellular Motility via Non-Uniform Temporal Sampling in Dynamic Optical Coherence Tomography

Amy L. Oldenburg,[a,b,*] Pan Ji,[a] Xiao Yu,[b] Lin Yang[a]
[a]University of North Carolina at Chapel Hill, Department of Physics and Astronomy, Chapel Hill, NC 27599-3255, USA
[b]University of North Carolina at Chapel Hill, Biomedical Research Imaging Center, Chapel Hill, NC 27599-7513, USA

1 Dependence of Motility Amplitude on Memory Time

The plots in Fig. S1 demonstrate in simulations (via Eq. (6) – (8)) how the memory time p (in terms of numbers of samples, where the total number of samples $N = 100$) affects the measurement of the motility amplitude, M. As p decreases, a more significant portion of the motile scatterer contributions decorrelate during the sampling time t_s, and the approximation of Eq. (10) breaks, leading to an under-estimate of $\Gamma(t_s)$ as shown in Fig. S1A. This, then, leads to an under-estimate of M relative to the underlying c_m when p is too short (Fig. S1c). At the other extreme, as the memory time is increased, in this case to up to half of t_{total} ($p = 50$), the approximation of Eq. (11) breaks and $\bar{S}_{OCT}{}^2$ becomes increasingly over-estimated. This also leads to an under-estimate of M. As such, there is a range of p over which M faithfully represents c_m. While a simplified model is employed here to show these general trends, the exact limits on p will depend upon the physics of the scatterer motion, error tolerance, and choices of t_s and N.

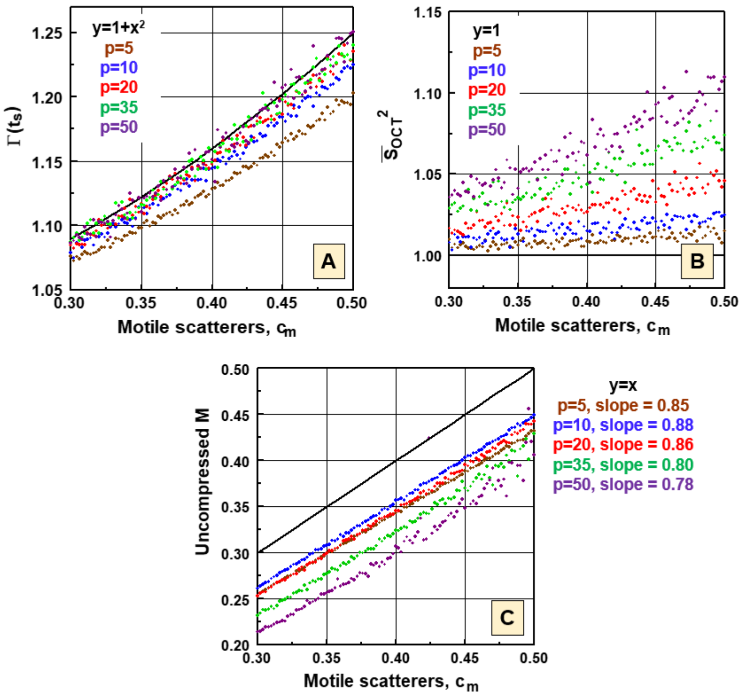

Fig. S1 Simulations of uncompressed M versus motile scatterer contribution c_m with varying memory times p The noise contribution was fixed at $c_n = 0.25$. (A) Plots of the first autocorrelation sample $\Gamma(t_s)$ show how it is under-estimated, relative to the ideal curve given by Eq. (10), as memory time is shortened. (B) Plots of $\bar{S}_{OCT}{}^2$ show how it is over-estimated as memory time is lengthened. (C) Corresponding plots of M, which depends on the difference between $\Gamma(t_s)$ and $\bar{S}_{OCT}{}^2$, show how the regression line slope is lower than the ideal $y=x$ line, with a lowered slope when the memory time is either too short or too long.

2 Pixel-Wise Analysis of NUTS in OCT of MEC Spheroids

In support of scatter plot data of *M* presented in Fig. 3, where the regression line slopes and correlation coefficients (Pearson's *r*) are reported for a select spheroid under varying levels of compression, in Table S1 we provide tabular data, averaged by culture condition, across all spheroids imaged.

Time	Dose (μM)	Slope			Pearson's r		
		2x CS	4x CS	8x CS	2x CS	4x CS	8x CS
Before	0	0.98	0.96	0.91	0.91	0.83	0.70
	25	0.98	0.95	0.91	0.92	0.83	0.71
	50	0.98	0.96	0.91	0.91	0.83	0.71
1 hour	0	0.98	0.96	0.91	0.93	0.84	0.72
	25	0.98	0.96	0.90	0.92	0.84	0.72
	50	0.98	0.96	0.91	0.93	0.84	0.72
24 hours	0	0.98	0.96	0.91	0.91	0.81	0.69
	25	0.98	0.95	0.90	0.91	0.82	0.69
	50	0.98	0.95	0.90	0.91	0.81	0.69
48 hours	0	0.98	0.96	0.91	0.89	0.79	0.67
	25	0.98	0.95	0.89	0.91	0.81	0.68
	50	0.97	0.94	0.89	0.88	0.78	0.65
6 days	0	0.97	0.94	0.88	0.88	0.76	0.62
	25	0.97	0.94	0.89	0.89	0.79	0.68
	50	0.97	0.94	0.90	0.89	0.80	0.69
Average		0.98	0.95	0.90	0.90	0.81	0.69

Table S1. Slope and Pearson's *r* of non-uniformly sampled (2-, 4-, and 8-fold compressed) versus uncompressed *M*.

3 Spheroid-Averaged Analysis of NUTS in OCT

To support data presented in Fig. 4B showing general results of hypothesis testing on uncompressed and non-uniformly sampled *M*, in Table S2 below we provide tabular data of spheroid-averaged *M* grouped by each condition and compression method, then, resulting p-values computed by comparing *M* for each group against its baseline (before) value. P-values considered to be significant are highlighted in yellow.

Time	Dose (μM)	M (mean ± std err)			p value		
		Uncompressed	4x CS	8x CS	Uncompr	4x CS	8x CS
Before	0	0.289 ± 0.004	0.277 ± 0.004	0.263 ± 0.004			
	25	0.270 ± 0.005	0.259 ± 0.005	0.246 ± 0.004			
	50	0.275 ± 0.005	0.263 ± 0.005	0.250 ± 0.005			
1 hour	0	0.291 ± 0.005	0.280 ± 0.005	0.266 ± 0.005	0.83	0.73	0.64
	25	0.283 ± 0.004	0.271 ± 0.004	0.256 ± 0.004	0.042	0.049	0.093
	50	0.279 ± 0.004	0.268 ± 0.004	0.255 ± 0.004	0.52	0.48	0.44
24 hours	0	0.279 ± 0.006	0.267 ± 0.006	0.254 ± 0.005	0.12	0.14	0.18
	25	0.260 ± 0.005	0.249 ± 0.005	0.236 ± 0.005	0.19	0.17	0.12
	50	0.252 ± 0.004	0.240 ± 0.004	0.227 ± 0.004	1.3 E-3	5.0 E-4	3.1 E-4
48 hours	0	0.285 ± 0.01	0.274 ± 0.008	0.260 ± 0.009	0.70	0.79	0.81
	25	0.272 ± 0.005	0.258 ± 0.004	0.243 ± 0.004	0.73	0.92	0.61
	50	0.231 ± 0.004	0.219 ± 0.004	0.208 ± 0.004	3.7 E-9	2.9 E-10	1.0 E-10
6 days	0	0.292 ± 0.006	0.275 ± 0.007	0.258 ± 0.006	0.75	0.76	0.53
	25	0.233 ± 0.004	0.220 ± 0.004	0.209 ± 0.004	1.4 E-8	2.6 E-9	5.3 E-10
	50	0.212 ± 0.005	0.202 ± 0.005	0.192 ± 0.004	7.9 E-14	8.2 E-15	4.6 E-15

Table S2 Results of hypothesis testing on blebbistatin-treated MEC spheroids as a function of compression (either uncompressed, or 4- or 8-fold compressed by non-uniform sampling).

4 Results of hypothesis testing for uniform temporal sub-sampling

Below are results of UTS on the spheroid data, plotted in the same way as results of NUTS in Fig. 4 of the manuscript.

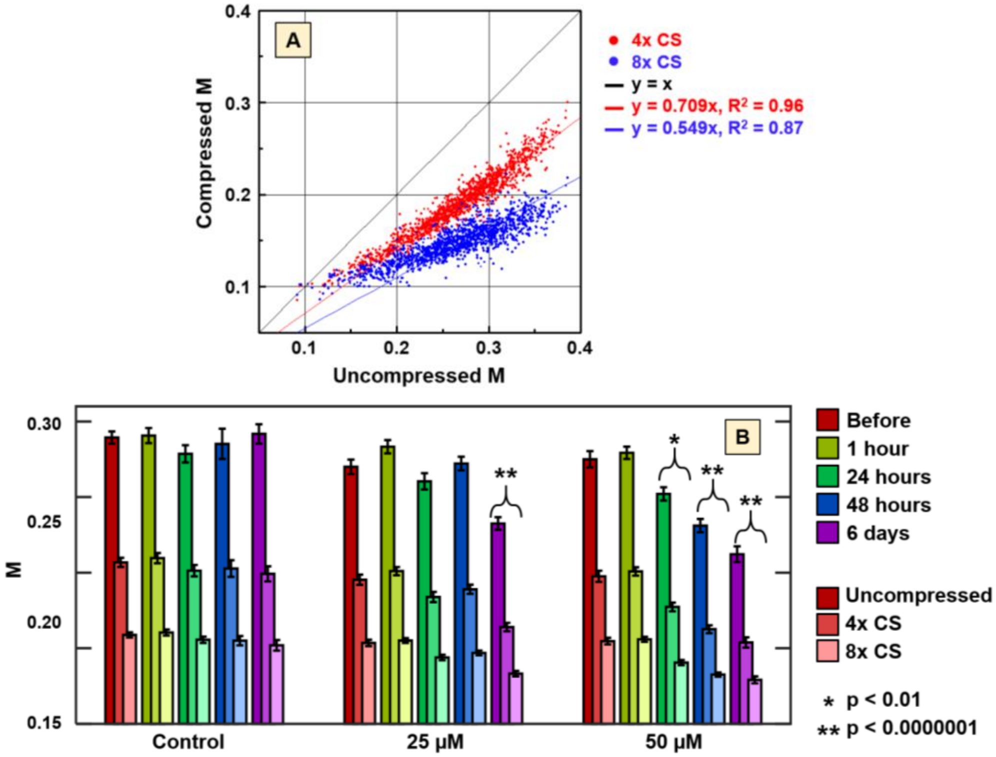

Fig. S2 Computations of uniformly sampled *M*, displayed in the same way as non-uniformly sampled *M* data of Fig. 4. (A) Spheroid-by-spheroid scatter plot of 4× and 8× UTS versus uncompressed *M* over all blebbistatin concentrations and time points (*n*=1428). (B) Dose- and time-dependent *M* (mean ± std. err) of spheroids exposed to blebbistatin under varying compression levels, with results of hypothesis testing (2-tailed t-test compared to corresponding before values).
